# Interaction between Simian Virus 40 Major Capsid Protein VP1 and Cell Surface Ganglioside GM1 Triggers Vacuole Formation

**DOI:** 10.1128/mBio.00297-16

**Published:** 2016-03-22

**Authors:** Yong Luo, Nasim Motamedi, Thomas G. Magaldi, Gretchen V. Gee, Walter J. Atwood, Daniel DiMaio

**Affiliations:** a Department of Genetics, Yale School of Medicine, New Haven, Connecticut, USA; b Department of Molecular Biology, Cell Biology, and Biochemistry, Brown University, Providence, Rhode Island, USA; c Department of Molecular Biophysics and Biochemistry, Yale School of Medicine, New Haven, Connecticut, USA; d Department of Therapeutic Radiology, Yale School of Medicine, New Haven, Connecticut, USA; e Yale Cancer Center, New Haven, Connecticut, USA

## Abstract

Simian virus 40 (SV40), a polyomavirus that has served as an important model to understand many aspects of biology, induces dramatic cytoplasmic vacuolization late during productive infection of monkey host cells. Although this activity led to the discovery of the virus in 1960, the mechanism of vacuolization is still not known. Pentamers of the major SV40 capsid protein VP1 bind to the ganglioside GM1, which serves as the cellular receptor for the virus. In this report, we show that binding of VP1 to cell surface GM1 plays a key role in SV40 infection-induced vacuolization. We previously showed that SV40 VP1 mutants defective for GM1 binding fail to induce vacuolization, even though they replicate efficiently. Here, we show that interfering with GM1-VP1 binding by knockdown of GM1 after infection is established abrogates vacuolization by wild-type SV40. Vacuole formation during permissive infection requires efficient virus release, and conditioned medium harvested late during SV40 infection rapidly induces vacuoles in a VP1- and GM1-dependent fashion. Furthermore, vacuolization can also be induced by a nonreplicating SV40 pseudovirus in a GM1-dependent manner, and a mutation in BK pseudovirus VP1 that generates GM1 binding confers vacuole-inducing activity. Vacuolization can also be triggered by purified pentamers of wild-type SV40 VP1, but not by GM1 binding-defective pentamers or by intracellular expression of VP1. These results demonstrate that SV40 infection-induced vacuolization is caused by the binding of released progeny viruses to GM1, thereby identifying the molecular trigger for the activity that led to the discovery of SV40.

## IMPORTANCE

The DNA tumor virus SV40 was discovered more than a half century ago as a contaminant of poliovirus vaccine stocks, because it caused dramatic cytoplasmic vacuolization of permissive host cells. Although SV40 played a historically important role in the development of molecular and cellular biology, restriction mapping, molecular cloning, and whole-genome sequencing, the basis of this vacuolization phenotype was unknown. Here, we show that SV40-induced vacuolization is triggered by the binding of the major viral capsid protein, VP1, to a cell surface ganglioside receptor, GM1. No other viral proteins or virus replication is required for vacuole formation. Other polyomaviruses utilize different ganglioside receptors, but they do not induce vacuolization. This work identifies the molecular trigger for the phenotype that led to the discovery of this important virus and provides the first molecular insight into an unusual and enigmatic cytopathic effect due to virus infection.

## INTRODUCTION

In the late 1950s, Sweet and Hilleman observed that incubation of African green monkey kidney cells with poliovirus vaccine stocks caused dramatic cytoplasmic vacuolization (1). They discovered that this activity was due to a contaminating lytic monkey virus, which they isolated and named simian vacuolating virus, a virus now known as simian virus 40 (SV40). As well as replicating productively in monkey cells, SV40 can cause tumors in hamsters (2). This finding raised concern that SV40 may cause cancer in humans, because it had been inadvertently inoculated into millions of people undergoing poliovirus vaccination. However, current evidence indicates that SV40 has little if any pathogenicity in humans, a fortuitous outcome that was by no means certain when the virus was first isolated (3, 4). In the decades since its discovery, studies of SV40 led to important insights into basic mechanisms of numerous cell processes, including gene transcription, DNA replication, and cell transformation (5). In addition, tools developed to study SV40 that were later adapted to study cellular genes include restriction mapping, molecular cloning, site-directed mutagenesis, and whole-genome sequencing (6–10).

SV40 belongs to the *Polyomaviridae* family, a group of small, nonenveloped, double-stranded DNA viruses that also includes pathogenic human viruses such as BK virus (BKV), JC virus, and the most recently discovered human tumor virus, Merkel cell polyomavirus (11). The icosahedral SV40 capsid consists of 360 molecules of the major capsid protein VP1, which assemble into 72 pentamers that form the outer capsid shell (12). Two closely related minor capsid proteins, VP2 and VP3, are localized to the interior of the capsid.

Dramatic cytoplasmic vacuolization is the most distinguishing feature of lytic SV40 infection of African green monkey kidney cells and certain other permissive monkey cells. SV40 is the only known polyomavirus with significant vacuolizing activity. However, 56 years after the discovery of SV40, the mechanism of SV40-induced vacuolization is not known. Vacuolization normally occurs late during infection, coincident with the release of progeny virus. In addition, transient, early cellular vacuolization (ECV) occurs in primary African green monkey kidney cells infected at a high input multiplicity by infectious or UV-inactivated SV40, suggesting the capacity to induce early vacuolization resides in a structural component of the SV40 virion and does not require replication of SV40 DNA (13). The relationship between ECV and classic vacuolization at late times is not known. Neither the viral nor the cellular components required for early or late vacuolization have been identified.

The cell surface ganglioside GM1 is the receptor for SV40, while other polyomaviruses utilize different gangliosides for virus binding and entry (14–17). The carbohydrate moiety of these gangliosides binds directly to binding pockets generated by pentamerization of VP1 (18). The pentameric nontoxic B subunit of cholera toxin (CTXB) also binds to GM1 (19). Helenius and coworkers showed that binding of SV40 capsids or isolated pentameric VP1 to cell surface GM1 induced dramatic membrane curvature that led to the formation of tight-fitting, virus-filled invaginations and tubules of the plasma membrane of monkey CV-1 cells (20). This process appears to provide a route of virus internalization during cellular uptake of SV40.

Amino acid substitutions in VP1 can alter the receptor specificity of polyomaviruses (16, 21). Previously, by introducing mutations in the GM1 binding site on SV40 VP1, we and others have isolated viable mutant viruses that no longer use GM1 for infection but appear to use alternative ganglioside receptors (21, 22). These mutations also inhibit the ability of SV40 to induce vacuolization in permissive monkey cells without impairing virus replication, raising the possibility that the VP1-GM1 interaction plays a role in mediating vacuolization. Here, we show that SV40-induced vacuolization is triggered by the massive amount of released progeny viruses during the late stage of permissive infection and the resulting binding of higher-order VP1 pentameric structures to GM1 at the cell surface.

## RESULTS

### SV40 mutants defective for GM1 binding fail to induce vacuole formation in CV-1 cells.

**FIG 1:**
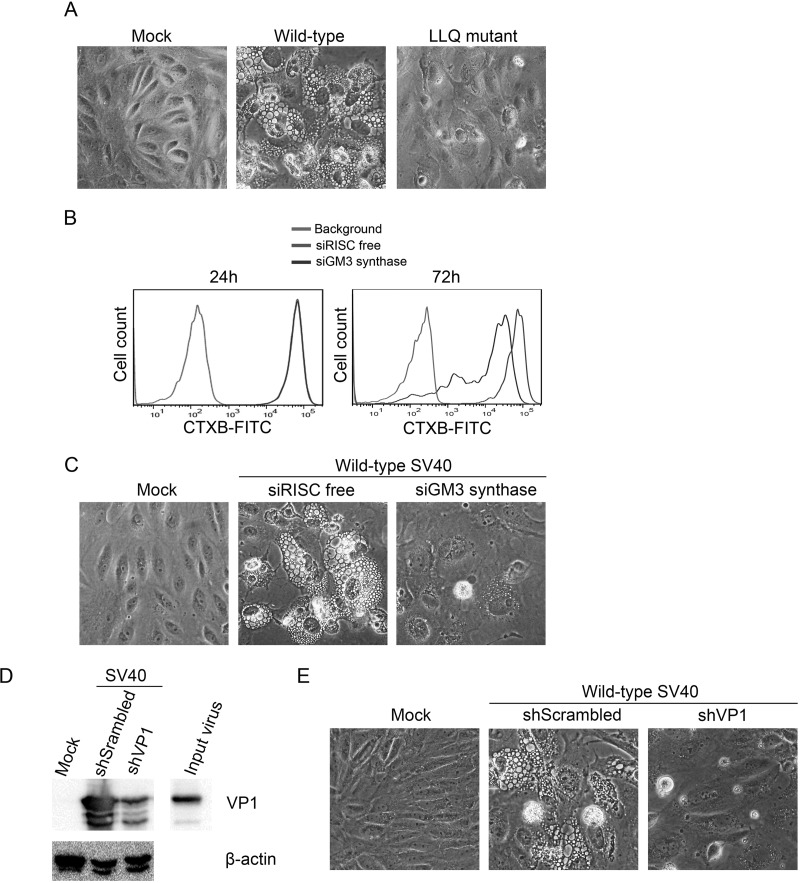
Interference with GM1-VP1 binding blocks vacuolization. (A) CV-1 cells were infected at an MOI of 10 with wild-type SV40 or a GM1 binding-defective mutant containing three mutations (A70L/F75L/H129Q) in VP1 (LLQ mutant). Phase-contrast images were taken 72 h postinfection. (B and C) CV-1 cells were transfected with GM3 synthase siRNA or a control RISC-free siRNA. After 24 and 72 h, cell surface GM1 was analyzed by staining unpermeabilized cells with fluorescently labeled CTXB followed by flow cytometry (B). At 24 h posttransfection, cells were infected with wild-type SV40 at an MOI of 10. Phase-contrast images were taken at 72 h postinfection (C). (D and E) CV-1 cells were infected with lentivirus expressing shRNA targeting SV40 VP1 or a scrambled control. Two days later, cells were infected with wild-type SV40 at an MOI of 10. VP1 levels were determined by Western blot analysis at 72 h after infection. β-Actin was used as a loading control. Input virus was used as a control (D). Phase-contrast images were taken 72 h after SV40 infection (E).

Ganglioside GM1 is the cell surface receptor that mediates entry of wild-type SV40 during permissive infection (14, 15). Previously, we identified several viable SV40 mutants that were defective for GM1 binding because of amino acid substitutions in the GM1 binding site of VP1 (21). Interestingly, these mutants replicate well (apparently by utilizing a different ganglioside to support infection) but fail to induce vacuole formation in permissive CV-1 monkey cells. An SV40 mutant partially defective for GM1 binding was also partially defective for vacuole induction, and Murata and colleagues reported a different mutation in the GM1 binding site of VP1 that appeared to affect GM1 binding and also prevented vacuolization (21, 23). Here, we focused on the VP1 mutant that has the lowest GM1 binding ability. This mutant contains 3 amino acid substitutions, alanine 70 to leucine, phenylalanine 75 to leucine, and histidine 129 to glutamine, that appear to occlude the GM1 binding site (designated here the LLQ mutant) (21). As shown in Fig. 1A, many CV-1 cells infected at a multiplicity of infection (MOI) of 10 with wild-type SV40 but not the LLQ mutant displayed dramatic vacuolization 72 h postinfection. It is important to note that the wild-type and mutant viruses entered cells and replicated to similar extents with the same kinetics, as shown by large T-antigen expression levels at different times postinfection (see Fig. S1A in the supplemental material). In addition, a second GM1 binding-defective VP1 mutant with a similar vacuolization-defective phenotype accumulated abundant virus particles in the nucleus of infected cells, confirming that these mutants do not display an entry or replication defect (21) (see Fig. S1B). These results suggest that the ability of SV40 to induce vacuole formation is related to GM1 binding.

To confirm the role of GM1 in triggering vacuolization, we performed small interfering RNA (siRNA) transfection to knock down GM3 synthase, an enzyme required for the synthesis of GM3, the precursor of GM1. Cell surface GM1 levels were not affected at 24 h post-siRNA transfection but were depleted approximately 4-fold by 72 h posttransfection, as assessed by flow cytometry for CTXB binding (Fig. 1B). Wild-type SV40 was inoculated at an MOI of 10 at 24 h post-siRNA transfection to ensure that virus entry occurred prior to the reduction in cell surface GM1. Efficient infection of the control and knockdown cells was confirmed by T antigen staining and flow cytometry (see Fig. S2 in the supplemental material). At 72 h postinfection, vacuole formation was almost completely abrogated in siRNA-GM3-transfected cells, but not in cells transfected with the siRNA control (Fig. 1C). This result provided further evidence that GM1 is required for SV40-induced vacuolization. In addition, it suggested that the initial interaction between GM1 and VP1 during virus entry at this relatively low MOI is not sufficient for vacuolization.

Similarly, we depleted VP1 synthesis by lentivirus-mediated shRNA transduction during SV40 infection. VP1 levels at late times in infection were depleted to an amount similar to that present in the input virus (Fig. 1D). As shown in Fig. 1E, VP1 knockdown caused a striking inhibition of vacuole formation, demonstrating that VP1 is required for host cell vacuolization. We note that this RNA interference approach inhibits only new VP1 synthesis occurring at late times during SV40 infection. This finding implies that vacuolization is mediated by newly synthesized and not input VP1, and again that the GM1-VP1 interaction during virus entry at a relatively low MOI is not sufficient for vacuolization. Taken together, these results suggest that binding of newly synthesized VP1 to GM1 plays a key role in inducing host cell vacuolization.

### Intracellular expression of VP1 does not cause vacuole formation in CV-1 cells.

**FIG 2:**
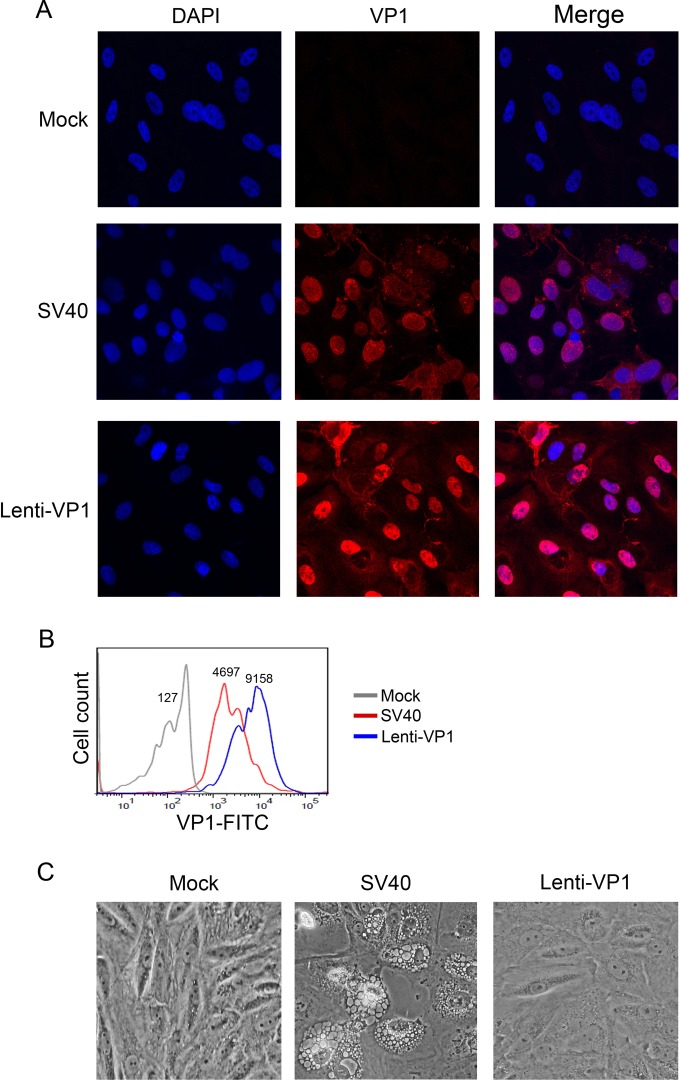
Ectopic expression of VP1 does not induce vacuolization. CV-1 cells were infected with SV40 (MOI, 10) or with lentivirus expressing SV40 VP1. (A) At 72 h after infection, cells were fixed, permeabilized, stained with anti-VP1 antibody PAb597 (red) and DAPI (4′,6-diamidino-2-phenylindole; blue), and visualized by fluorescence microscopy. The panels in each row show the same field with a merged fluorescent image shown on the right. (B) Cells harvested 72 h postinfection were analyzed by immunostaining with PAb597 followed by flow cytometry to measure VP1 expression levels. Mean fluorescent intensity values are shown above the individual histograms. (C) Cells were photographed by using phase-contrast microscopy 72 h after infection.

Since VP1 is required for vacuolization, we tested whether overexpression of VP1 *per se* induces vacuoles in CV-1 cells. Lentivirus-mediated ectopic expression of wild-type SV40 VP1 resulted in VP1 expression levels equal to or higher than those during the late stages of productive SV40 infection, as assessed by immunofluorescence (IF) staining and by flow cytometry (Fig. 2A and B). As expected, ectopically expressed VP1 was localized largely to the nucleus. Despite this high expression level, ectopic VP1 failed to induce vacuole formation in CV-1 cells (Fig. 2C). Thus, nuclear expression of VP1 fails to cause vacuolization, suggesting that GM1-VP1 binding at the cell surface is required for vacuolization and/or that vacuolization requires virus replication or viral gene products in addition to VP1.

### Vacuolization during productive SV40 infection is caused by released progeny viruses that bind to GM1.

**FIG 3:**
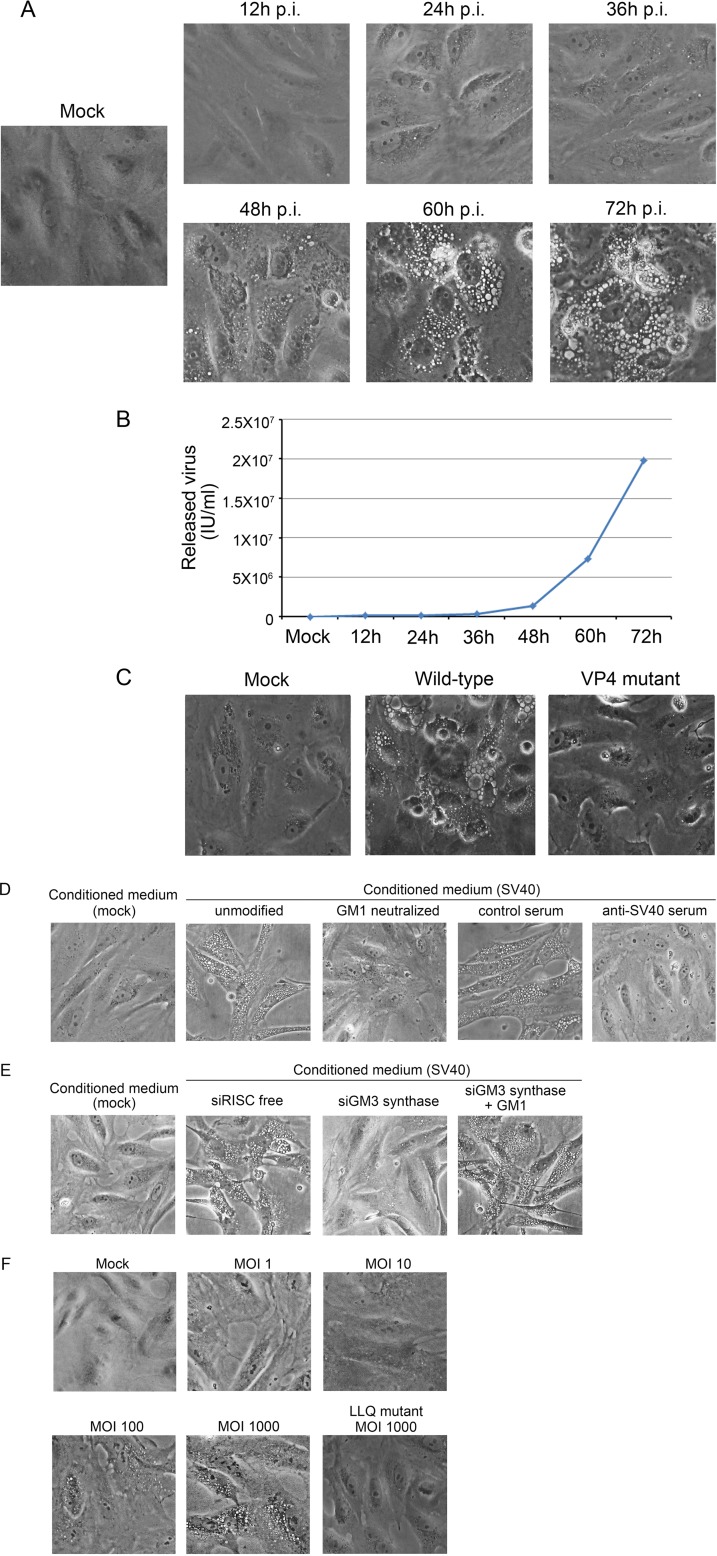
SV40 infection-induced vacuolization is caused by reinfection by released progeny viruses. (A and B) CV-1 cells were infected with SV40 at an MOI of 10. Phase-contrast images were taken at the indicated time points postinfection (A). In a parallel experiment, cell culture medium was collected and the number of released viruses per milliliter of culture medium was quantified by infecting naive CV-1 cells followed by flow cytometry to enumerate T antigen-positive cells (B). (C) CV-1 cells were mock infected or infected with wild-type SV40 or VP4 mutant at an MOI of 10. At 72 h after infection, phase-contrast images were acquired. Mock-infected cells were used as a control. (D) CV-1 cells were mock infected or infected with wild-type SV40 at an MOI of 10. At 72 h postinfection, the conditioned medium was collected and incubated with uninfected CV-1 cells. As indicated, conditioned medium was incubated with soluble GM1 or with control or neutralizing anti-SV40 serum. Phase-contrast images were taken 4 h after addition of conditioned medium. (E) CV-1 cells transfected with control RISC-free siRNA or with siRNA targeting GM3 synthase. 72h later, cells were treated with conditioned medium from infected or mock-infected CV-1 cells. As indicated, 16 h prior to incubation with conditioned medium, 5 µM GM1 was added to cells transfected with the siRNA targeting GM3 synthase. Phase-contrast images were taken 4 h after addition of conditioned medium. (F) CV-1 cells were mock infected or infected with wild-type SV40 at the indicated MOIs or the LLQ mutant at an MOI of 1,000. Phase-contrast images were obtained 4 h later.

During SV40 infection at an MOI of 10, vacuole formation occurred at the late stage of infection (60 to 72 h postinfection) (Fig. 3A). Determination of titers of virus released into the cell culture medium at different time points postinfection showed a dramatic increase of released progeny virus at these late times (Fig. 3B). Compared with the initial infection with an input of 10 infectious units (IU)/cell, massive numbers of progeny viruses (~1,000 IU/cell) were released into the medium by 60 h postinfection, approximately the time when large numbers of vacuoles formed. Therefore, we hypothesized that vacuolization during productive infection is caused by progeny viruses binding to GM1.

To test the role of released virus in vacuolization occurring late during infection, we determined whether vacuolization was abrogated by reducing the release of progeny viruses from infected cells. SV40 release is mediated in part by the late viral protein VP4, which forms pores in infected cell membranes (24, 25). Although the absence of VP4 inhibits SV40 release, it has little influence on intracellular viral replication or VP1 expression (24, 26). We infected CV-1 cells with wild-type and VP4 mutant SV40 at an MOI of 10 and confirmed that the VP4 mutation impaired progeny virus release despite robust intracellular replication of the mutant (see Fig. S3 in the supplemental material). In parallel, we compared the ability of the VP4 mutant and wild-type SV40 viruses to induce vacuole formation. As shown in Fig. 3C, the absence of VP4 caused substantial inhibition of vacuole formation.

As an additional test of the ability of progeny virus to induce vacuoles, we treated CV-1 cells with conditioned medium collected 72 h postinfection of CV-1 cells by wild-type SV40. Following treatment with conditioned medium from infected cells, vacuolization occurred within 4 h (Fig. 3D), a time prior to the initiation of SV40 replication as assessed by the lack of large T antigen expression (see Fig. S4 in the supplemental material). Importantly, the acute vacuolization induced by conditioned medium was blocked by neutralizing SV40 antiserum (Fig. 3D), by soluble GM1, which binds to authentic SV40 and neutralizes infection (21) (Fig. 3D), or by knockdown of GM3 synthase in the recipient cells (Fig. 3E). The vacuolization defect in the knockdown cells was rescued by addition of GM1 to the culture medium, which inserts into the plasma membrane of the cells, where it can serve as the SV40 receptor (15) (Fig. 3E). Thus, conditioned medium from SV40-infected cells contains a factor that acutely induces vacuolization in a VP1- and GM1-dependent fashion. Similarly, acute vacuolization occurred when CV-1 cells were infected with wild-type SV40 at a high MOI (100 to 1,000), whereas the LLQ mutant was defective for inducing vacuoles even at an MOI of 1,000 (Fig. 3F). Taken together, these results strongly suggest that vacuolization during SV40 permissive infection is caused by the massive amount of released progeny viruses that bind to GM1.

### Vacuolization is triggered by GM1 binding but not virus replication.

**FIG 4:**
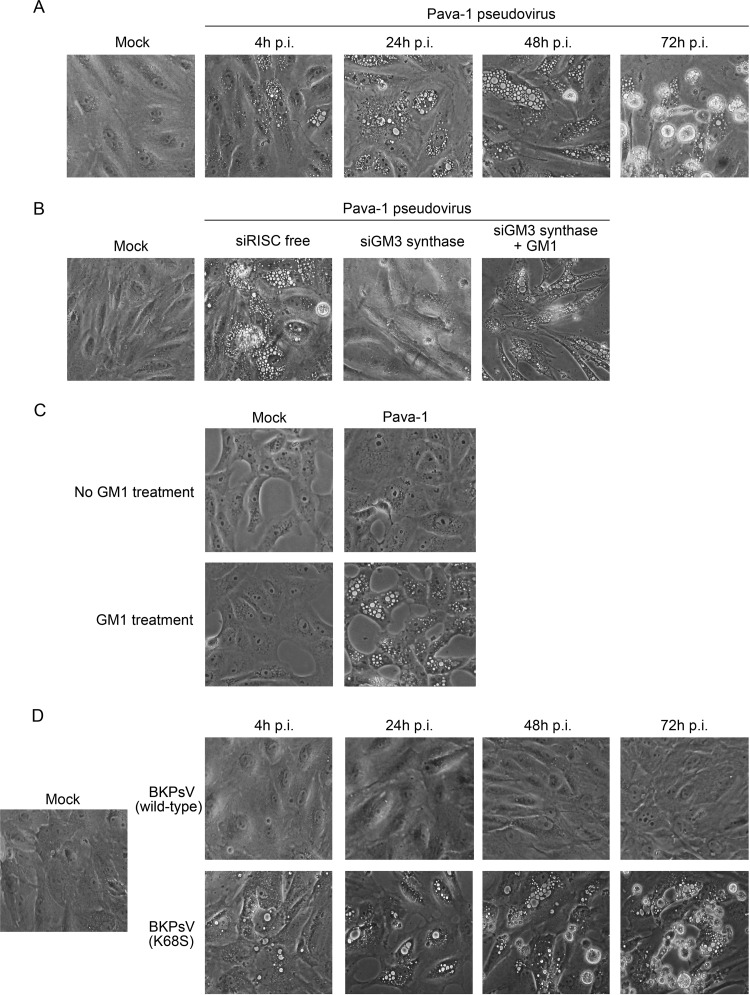
Pava-1 and GM1-binding BKpsV induce acute vacuolization. (A) CV-1 cells were infected with Pava-1 at an MOI of 1,000. Phase-contrast images were taken at the indicated time points postinfection; mock-infected cells at the 72-h time point were used as a control. (B) CV-1 cells were transfected with a control RISC-free siRNA or siRNA targeting GM3 synthase. At 72 h posttransfection, cells were infected with Pava-1 at an MOI of 1,000. To rescue GM1 depletion, cells transfected with GM3 synthase siRNA as described in the text were treated with 5 µM GM1 14 h prior to Pava-1 infection. Phase-contrast images were taken at 4 h postinfection. Mock-infected cells were used as a control. (C) Vero cells were pretreated with 10 µM GM1 for 16 h or left untreated. Cells were then infected with Pava-1 at an MOI of 1,000 or mock infected. Phase-contrast images were acquired 4 h later. (D) CV-1 cells were infected with 10^5^ genome copies/cell of wild-type BK pseudovirus or BKpsV containing a K68S mutation in VP1 that generates binding to GM1. Phase-contrast images were taken at the indicated times points postinfection; mock-infected cells at 72 h were used as a control.

The experiments described above indicate that SV40 released at late times in infection induces vacuolization in a GM1-dependent fashion. Furthermore, the ability of SV40 or progeny virus in conditioned medium to rapidly induce vacuolization in naive CV-1 cells implies that neither virus replication nor a late infected cell milieu is required in the cells undergoing vacuolization. To establish conclusively that virus replication is not required for vacuolization, we infected CV-1 cells with a replication-defective SV40 pseudovirus named Pava-1. Pava-1 consists of the SV40 capsid surrounding a genome that cannot replicate in CV-1 cells because the SV40 large T antigen gene, which is absolutely required for SV40 DNA replication, has been replaced by a segment of bovine papillomavirus type 1 (BPV) DNA encoding the BPV E2 transcription factor and the E5 oncoprotein (27). As shown in Fig. 4A, at an MOI of 1,000 Pava-1 caused vacuolization in CV-1 cells beginning as early as 4 h postinfection. Vacuoles increased in size with increasing time postinfection, and significant cell death was evident at 72 h. Acute vacuolization by Pava-1 was inhibited by knockdown of GM3 synthase and restored by addition of GM1 to the knockdown cells (Fig. 4B). This result indicates that a large number of Pava-1 particles can cause acute GM1-dependent vacuolization in the absence of viral DNA replication and also that persistent vacuolization can occur in cells that are not undergoing events occurring late in the lytic virus life cycle.

We also tested the vacuolization activity of Pava-1 in Vero cells, an African green monkey kidney cell line with lower cell surface GM1 levels than CV-1 cells (see Fig. S5 in the supplemental material). Pava-1 infection of unmodified Vero cells failed to induce vacuolization, consistent with the low level of endogenous GM1 in these cells, but incorporation of GM1 into the plasma membrane allowed robust vacuolization (Fig. 4C; see also Fig. S5). Thus, acute vacuolization induced by a replication-defective SV40 pseudovirus is GM1 dependent, as is classic vacuolization occurring late during productive virus replication.

As an independent approach to determine whether GM1 binding is required for acute vacuole formation in the absence of virus replication, we studied the related human polyomavirus BKV. Although the VP1 sequences of SV40 and BKV are very similar and are thought to engage their ganglioside receptors in a similar fashion, wild-type BKV utilizes ganglioside GD1b or GT1b for infection but does not bind to GM1 and fails to induce vacuolization (16, 18, 28). For the experiments reported here, we used CsCl-purified replication-defective BK pseudoviruses (BKpsV), consisting of a Gluc reporter genome packaged in a capsid composed of BKV VP1 and VP2/3 (16). As expected because of its inability to bind to GM1, wild-type BKpsV did not induce vacuolization in CV-1 cells (Fig. 4D). We previously reported a single amino acid substitution in the ganglioside binding site of BKV VP1, lysine 68 to serine (K68S), which allows the mutant VP1 pentamers to bind to GM1 (16). Strikingly, unlike wild-type BKpsV, the K68S BKpsV mutant induced acute and persistent vacuolization in CV-1 cells, as well as cell death at late times (Fig. 4D). Wild-type and K68S BKpsV infected CV-1 cells to a comparable extent, as assessed by expression of luciferase from the packaged reporter genome (see Fig. S6 in the supplemental material). Collectively, these results demonstrate that vacuolization can be acutely induced by VP1-GM1 binding even in the absence of virus replication and that the inability of BKV to induce vacuolization is due solely to its inability to bind to GM1.

### SV40 VP1 pentamers and cholera toxin B induce acute vacuolization.

**FIG 5:**
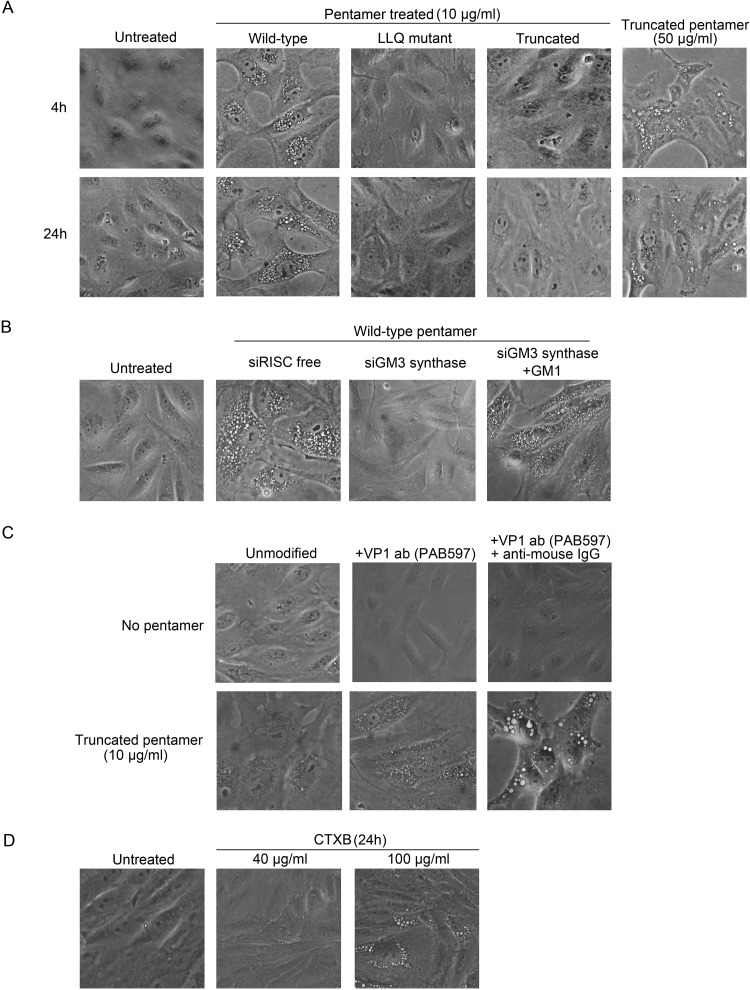
SV40 pentamers induce acute vacuolization. (A) CV-1 cells were treated with 10 µg/ml of the indicated SV40 VP1 pentamers or with 50 µg/ml ΔC truncated pentamers, and phase-contrast images were taken at 4 h and 24 h posttreatment. (B) CV-1 cells were transfected with a control RISC-free siRNA or siRNA targeting GM3 synthase. At 72 h posttransfection, cells were treated with 10 µg/ml wild-type pentamers. To rescue GM1 depletion, cells transfected with GM3 synthase siRNA as described in the text were then treated with 5 µM GM1 14 h prior to pentamer treatment. Phase-contrast images were taken 4 h post-pentamer treatment. Untreated cells were used as a control. (C) CV-1 cells were left untreated (top row) or treated with 10 µg/ml ΔC pentamers (bottom row). Prior to addition to cells, pentamers or mock solution was left untreated, preincubated with a 1:100 dilution of anti-VP1 (PAb597), or preincubated with anti-VP1 plus a 1:100 dilution of anti-mouse IgG, as indicated. Phase-contrast images were taken 4 h later. (D) CV-1 cells were treated with the indicated concentration of CTXB or with vehicle control. Phase-contrast images were taken 4 h later.

The pseudovirus stocks used in the preceding section contained not only VP1 but also VP2/3, as well as products released from the cells used to package pseudovirus. To determine if VP1 alone is able to induce acute vacuolization in the absence of other viral proteins and mammalian cell constituents, we used VP1 pentamers purified from bacteria. Bacterially produced SV40 VP1 oligomerizes to form pentamers and can assemble into higher-order pentameric structures, including virus-like particles (VLPs) (14, 29, 30). To determine whether VP1 pentamers are sufficient to induce vacuolization, we purified full-length wild-type SV40 VP1 from *Escherichia coli*. In parallel, we also purified GM1 binding-defective VP1 (containing the LLQ mutations) and a C-terminal deletion VP1 mutant (ΔC) that forms pentamers but is unable to form higher-order structures because the deletion removes sequences required for interpentameric interaction (18, 21). The ΔC pentamers have intact GM1 binding sites and remain competent for GM1 binding (21). Purified VP1 and its derivative mutants following SDS-PAGE are shown in Fig. S7A in the supplemental material. Electron microscopy (EM) confirmed that both wild-type VP1 and the ΔC mutant formed pentamers (see Fig. S7B). The full-length wild-type pentamers tended to form higher-order structures, but we did not observe intact VLPs in our preparations. In contrast, as expected, the ΔC pentamers did not associate with each other and remained individual pentamers. As shown in Fig. 5A, 10 µg/ml of full-length wild-type VP1 pentamers acutely induced vacuole formation in CV-1 cells. Vacuoles induced by pentamers tended to be smaller and more uniform in size than vacuoles induced by SV40 or pseudoviruses. Pentamer-induced vacuoles persisted for 24 h but were less evident at later time points (see Fig. S8 in the supplemental material). In contrast, the GM1 binding-defective pentamers did not induce vacuoles, even when tested at higher concentration (Fig. 5A; see also Fig. S7C). Furthermore, the ability of wild-type VP1 pentamers to induce vacuolization was inhibited by knockdown of GM3 synthase and rescued by addition of GM1 to the cells (Fig. 5B). These results confirm that the GM1-VP1 interaction is responsible for acute vacuolization and that viral replication and other viral or cellular proteins (e.g., VP2/3 or VP4) are not required.

The ΔC truncated pentamers at 10 µg/ml did not induce vacuoles, but vacuoles were induced when 50 µg/ml of the truncated pentamers were added to cells (Fig. 5A). To test whether the reduced ability of truncated VP1 to induce vacuole formation is due to its inability to form higher-order structures than pentamers, we used a nonneutralizing VP1 monoclonal antibody and secondary anti-IgG to cross-link ΔC pentamers prior to incubation with cells. As shown in Fig. 5C, cross-linked ΔC pentamers induced vacuolization in CV-1 cells as early as 4 h posttreatment, whereas antibody treatment in the absence of pentamers did not induce vacuoles. Pentamers cross-linked with anti-VP1 plus secondary anti-IgG were more effective at inducing vacuoles than pentamers treated with anti-VP1 alone. This result demonstrates that the truncated pentamers do not have an intrinsic vacuolization defect, but rather suggests that higher-order pentameric VP1 structures are more effective than individual pentamers at inducing vacuolization.

Like VP1, CTXB forms a pentameric structure that binds to GM1 (31). X-ray crystallographic studies demonstrated that CTXB and VP1 bind to GM1 in essentially the same conformation, but none of the specific molecular interactions responsible for binding is common to the two (18, 31). Figure 5D shows that CTXB also induced vacuolization in CV-1 cells, but at a 4- to 10-fold-higher concentration than that required for wild-type VP1 pentamers. These results show that vacuolization of CV-1 cells can be triggered by different pentameric structures that bind to GM1 and that no virus-specific sequences are required.

### VP1 does not accumulate in vacuoles.

**FIG 6:**
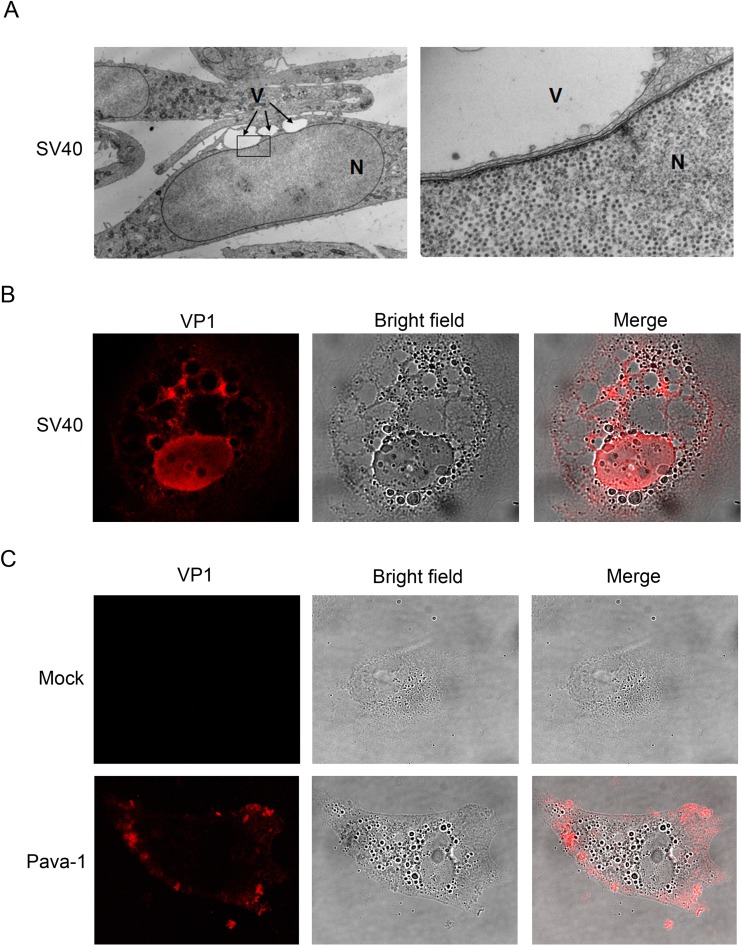
Progeny virions and VP1 do not accumulate in vacuoles. (A) CV-1 cells were infected with wild-type SV40, and thin sections were prepared after 64 h and examined by transmission electron microscopy. (Left) Low-magnification view; (right) enlargement of the boxed area of the image on the left. Note the large numbers of virus particles in the nucleus but not the vacuoles. V, vacuole; N, nucleus. (B) CV-1 cells were immunostained for VP1 (red) 72 h postinfection with wild-type SV40 at an MOI of 10. Images were acquired on a Leica SP5 confocal microscope. (Left) VP1 staining; (middle) bright-field image; (right) merged image. (C) CV-1 cells were immunostained for VP1 (red) 4 h after mock infection or exposure to Pava-1 at an MOI of 1,000. Images were obtained as described for panel B.

The experiments described above demonstrate that binding of SV40 VP1 to GM1 triggers the formation of vacuoles. We used three approaches to determine if VP1 accumulates in vacuoles. First, we used transmission electron microscopy to examine SV40-infected CV-1 cells late in infection. As shown in Fig. 6A, although progeny virus particles were present in high numbers in the nuclei of infected cells, we did not observe viruses within the vacuoles. Second, we stained CV-1 cells for intracellular VP1 late during productive infection with wild-type SV40 (Fig. 6B). Although nuclear and cytoplasmic VP1 was readily detectable, VP1 was not present within the vacuoles. Finally, we stained CV-1 cells for intracellular VP1 4 h after high-multiplicity infection with Pava-1, at a time when cytoplasmic vacuoles were present. The immunofluorescent images in Fig. 6C show that input VP1 was readily apparent at the cell periphery, far from the vacuoles themselves. Thus, although VP1 triggers vacuole formation, it does not accumulate within the vacuoles either late during productive SV40 infection or acutely following treatment of cells with SV40 pseudovirus.

## DISCUSSION

Vacuolization, a hallmark of productive SV40 infection in African green monkey cells, is a mysterious phenotype that led to the discovery of the virus more than half a century ago. In this report, we show that vacuolization is triggered by binding of newly synthesized and released VP1 to cell surface GM1 at the late stages of SV40 infection. In addition, we show that vacuolization does not require active virus replication or viral gene products other than VP1. Our study not only identifies the trigger for the vacuolization phenotype characteristic of this historically important virus, but also reveals a novel cellular function of GM1, namely, the ability to induce vacuole formation.

This study identifies the viral and cellular components required for SV40-induced vacuolization. Oligomeric SV40 VP1 is the only viral protein required for vacuolization, and virus replication or late viral events in the cells undergoing vacuole formation are not required. The cellular ganglioside GM1 is also required for vacuolization. Our data further show that a direct interaction between VP1 pentamers and GM1 is required for vacuoles to form. This conclusion is supported by the observations that point mutations in the GM1 binding site of SV40 VP1 that prevent GM1 binding also abolish vacuole formation, that knockdown of ganglioside synthesis inhibits vacuolization (which is restored by treating the knockdown cells with GM1), and that Vero cells that are naturally resistant to vacuolization can be converted into susceptible cells by incorporation of GM1 into the plasma membrane. Most strikingly, a single amino acid substitution in the ganglioside binding site of BKV VP1 that generates GM1 binding also generates vacuolization activity. This result demonstrates that a nonvacuolating polyomavirus can be converted into a vacuolating one simply by acquiring the ability to bind to GM1. Thus, the VP1-GM1 interaction is essential for two distinct features of the virus life cycle, virus binding during cell entry and vacuole formation at late times.

Several pieces of data indicate that the GM1-VP1 interaction plays a direct role in vacuole formation and not that this interaction merely delivers the virus or VP1 into cells where it induces vacuoles in a GM1-independent fashion. The SV40 VP1 mutants defective for GM1 binding are also defective for vacuolization even though they efficiently infect and replicate in CV-1 cells. These viable mutants were selected for their ability to undergo productive replication resistant to GM1-mediated neutralization and rely on other gangliosides for entry (21). Second, vacuolization is inhibited by siRNA-mediated depletion of GM1 after infection is established. In addition, wild-type BKpsV does not induce vacuoles in CV-1 cells even though it efficiently enters these cells, and the ability of BKpsV K68S to induce vacuoles is not due to more efficient entry. Finally, the B subunit of cholera toxin, whose only known biochemical activity is binding to GM1 and has no other biological similarity to VP1, can also induce vacuoles.

Our experiments also shed light on the relationship between classic vacuolization occurring late during infection and acute vacuolization induced by a high multiplicity of infection. It was initially suggested that these were two different phenomena because of the timing and durability of vacuole formation and differences in vacuole size (13). Our results provide strong evidence that early and late vacuoles result from the same biological process. Most notably, both early and late vacuoles require binding of VP1 to GM1. In addition, although vacuoles acutely induced by VP1 pentamers are small and transient, high-multiplicity infection with SV40 and BKV (K68S) pseudoviruses can result in the rapid formation of large vacuoles that persist for several days. The low numbers of virus typically used in infection studies are not sufficient to induce acute vacuolization. Rather, several lines of evidence indicate that vacuole formation at late times after infection at a standard MOI occurs in response to massive amplification and release of the virus. First, vacuole formation is coincident with virus release at late times. Second, newly synthesized VP1 is required for vacuolization. Third, late vacuolization is inhibited by a VP4 mutation that blocks virus release. In addition, expression of intracellular VP1 is not sufficient for vacuole formation. Finally, released progeny virus in the culture medium is sufficient to rapidly induce vacuole formation through a VP1-GM1 interaction. These results indicate that VP1 must be released from the infected cells and bind to GM1 on the cell surface to trigger vacuolization. Thus, vacuoles formed late in infection are in fact an acute response to the binding of recently released virus to GM1.

Pentamerization of VP1 is required to generate the ganglioside binding sites (18), but higher-order structures appear to be more active in inducing vacuolization. SV40 capsids (or GM1 binding K68S BPV capsids) are most active, whereas purified SV40 pentamers (which, although they can aggregate into higher-order structures, do not form VLPs in our hands) induce relatively small and transient vacuoles. In contrast, the ΔC pentamers, which are unable to form higher-order oligomers but retain GM1 binding, induced vacuolization only at a high concentration. Importantly, these truncated VP1 pentamers acquired robust vacuole-inducing activity after antibody-mediated cross-linking, demonstrating that higher-order structures induce vacuole formation more efficiently than individual pentamers. It is possible that interpentameric contacts generate new structural motifs or a geometry that facilitates vacuole formation, or that the increased number of close-proximity contacts due to large assemblages of VP1 may contribute avidity effects important for vacuole formation. Although CTXB binds to GM1 with ~1,000-fold-higher affinity than VP1 pentamers, it induces vacuoles only at much higher concentrations than VP1 pentamers, perhaps reflecting in part its inability to form higher-order oligomers (18, 19).

Neither progeny viruses nor VP1 pentamers accumulate within vacuoles, suggesting that vacuoles do not form from the virus-lined membrane invaginations described by Ewers et al. (20). This conclusion is consistent with the ability of truncated VP1 pentamers to induce invaginations (20), even though they are defective at inducing vacuoles. Rather, our results suggest that vacuole formation is mediated by a downstream signaling pathway(s) triggered by GM1 binding. This signaling may be related to GM1-induced signaling in other systems, such as neuronal cells (32). GM1 signaling leading to vacuole formation appears to be initiated by some specific feature of the VP1-GM1 complex, because binding of other polyomaviruses to their cognate gangliosides does not induce vacuoles. Thus, there must be a difference in the structure or clustering of VP1-bound GM1 itself or the resulting signal(s), compared with other ganglioside-VP1 combinations that do not trigger vacuole formation. In addition, the interaction of polyomaviruses with cells can acutely induce mitogenic signaling that is independent of ganglioside binding (33).

Our results show that GM1 binding plays different roles in different stages of SV40 infection. During virus entry, engagement of VP1 with GM1 is necessary for virus internalization (15, 34), most likely by causing stable association of the virion with the cell surface and causing plasma membrane invagination as an initial step in endocytosis (20). In addition, as demonstrated here, late during productive infection VP1-GM1 binding triggers vacuolization, which does not appear to be a direct result of plasma membrane invagination. It is possible that vacuole formation is a consequence of productive SV40 infection without any impact on virus replication, but we have not ruled out the possibility that vacuolization provides a benefit to SV40. We note that BKpsV (K68S) but not wild-type BKpsV induces vacuolization and cell death several days after infection, suggesting that there may be a direct link between vacuole formation and cell death. Such a process may be related to vacuole-associated nonapoptotic cell death induced by some chemicals (35–37). If SV40-induced vacuolization or the responsible signaling pathway facilitates cell death, this in turn might enhance progeny virus release and spread. This model is consistent with the finding that vacuolization-defective mutants, including LLQ, display a plaque-forming defect (reference 22; T. G. Magaldi and D. DiMaio, unpublished results). It is clear that the role of GM1-VP1 binding and vacuolization in virus replication and cell biology warrants further investigation.

## MATERIALS AND METHODS

### Reagents.

Monosialoganglioside GM1 (containing α-5-*N*-acetyl-muraminic acid) from bovine brain and unlabeled cholera toxin B were purchased from Sigma-Aldrich Corp. (St. Louis, MO). Alexa Fluor 488-labeled CTXB was purchased from Invitrogen (Carlsbad, CA). Rabbit anti-SV40 VP1 antibody was purchased from Abcam (Cambridge, MA). Monoclonal anti-SV40 VP1 polyclonal antibody (PAb) 597 and monoclonal anti-SV40 large T antigen PAb108 were harvested from cultured hybridoma cells. Neutralizing anti-SV40 horse antiserum was a kind gift of James Pipas, University of Pittsburgh. Lipofectamine RNAi Max reagent, SimplyBlue SafeStain, and SuperSignal West Pico chemiluminescent substrate were purchased from Thermo, Fisher (Grand Island, NY). A DNeasy kit was purchased from Qiagen (Valencia, CA). IScript cDNA synthesis and iQ SYBR green supermix kits were purchased from Bio-Rad (Hercules, CA). TaqMan gene expression master mix was purchased from Applied Biosystems (Foster City, CA).

### Cell culture and virus infection.

CV-1 cells and Vero cells (both of African green monkey origin) were purchased from the American Type Culture Collection and maintained in Dulbecco’s modified Eagle’s medium (DMEM) supplemented with 10% fetal bovine serum (FBS), 100 units/ml penicillin-streptomycin, 10 mM l-glutamine, and 10 mM HEPES (pH 7.2) in 5% CO_2_ at 37°C. An SV40 mutation preventing expression of VP4 was constructed by converting the VP4 start codon to ATA by site-directed mutagenesis (24). SV40 (strain 776), the LLQ mutant (21), and VP4 mutant virus stocks were produced from cloned DNA as previously described (38). Briefly, monolayers of CV-1 or CMT4 cells in T75 flasks were infected at an MOI of 10 with seed virus stock. After 4 to 5 days, when significant cell death was observed, flasks were subjected to multiple rounds of freeze-thawing. Cellular debris was removed by centrifugation at 1,000 rpm for 5 min, and supernatants were filtered through 0.45-µm syringe filters, aliquoted, and stored at −80°C. The VP4 mutant was passaged serially to generate sufficient virus. Virus titers, expressed as the number of infectious units per milliliter, were determined by infecting cells with virus stocks followed by staining for intracellular SV40 large T antigen with antibody PAb108 and flow cytometry at 48 h postinfection. Unless specified otherwise, infection was performed at an MOI of 10 IU per cell. Virus was added directly to the medium of cells at ~80% confluence, and the medium containing virus was not replenished for the duration of the experiment. Pava-5′BΔS pseudovirus (designated here Pava-1) was produced as described above and titers were determined by flow cytometry using the Abcam antibody to stain VP1 in infected CMT4 cells (27, 38). BKpsV and BKpsV(K68S) pseudoviruses (Dunlop strain) were produced in 293TT cells from plasmids expressing human codon-optimized BKV virion proteins and purified by using CsCl gradient centrifugation as described elsewhere for JC pseudovirus (17, 39), and titers were determined by quantitative PCR for encapsidated genome copies with primers targeting the GLuc gene in the phGluc reporter plasmid: forward primer, 5′-ACGCCCAAGATGAAGAAGTT-3′; reverse primer, 5′-ACCCAGGAATCTCAGGAATG-3′; and iQ SYBR Green Supermix (Bio-Rad). Approximately 10^5^ genome copies of BKpsV were added per cell for infection studies.

### Microscopy.

Phase-contrast images were taken at 40× magnification by using a Canon PowerShot G10 camera directly attached to an Olympus CK30 microscope. Immunofluorescence and bright-field images were taken by using a Leica SP5 confocal microscope at 60× or 100× magnification. Images were merged by using LAS AF (Leica) software.

### Flow cytometry.

Cell surface GM1 levels were determined by staining with Alexa Fluor 488-conjugated CTXB as previously described (21). To stain intracellular VP1, cells were fixed with methanol, permeabilized, and stained with a 1:200 dilution of VP1 antibody (Abcam) and an anti-rabbit secondary antibody. Stained cells were analyzed on an LSR II cytometer (BD Biosciences) at the Flow Cytometry Core, Yale University. All flow cytometry data were analyzed using FACSDiva and FlowJo software (BD Biosciences).

### GM3 synthase knockdown by siRNA transfection.

The siRNA oligonucleotide was purchased from Thermo Scientific (Grand Island, NY). The following target sequence was used to knock down expression of the GM3 synthase gene (ST3 beta-galactoside alpha-2,3-sialyltransferase 5 [ST3GAL5]): CAAUGGCGCUGUUAUUUGA. Twenty nanomoles of siRNA was transfected in CV-1 cells by reverse transfection using the Lipofectamine RNAi MAX reagent according to the manufacturer’s instructions.

### Lentiviral plasmid construction and virus production and use.

For SV40 VP1 knockdown, SV40 VP1 shRNA (5′-CCGGGAAGGGACTTCCCAGATATTTCTCGAGAAATATCTGGGAAGTCCCTTCTTTTTG-3′) was inserted into the pLKO.1 shRNA vector (Addgene, Cambridge, MA). Lentivirus was generated and concentrated following the instructions provided by the manufacturer. Cells were incubated with a 1:10 dilution of concentrated lentivirus stock for 48 h at 37°C. For SV40 VP1 expression, the DNA coding sequences for SV40 VP1 were human codon optimized by GenScript USA Inc. (Piscataway, NJ) and inserted into the pLenti-CMV-IRES-GFP-WPRE expression vector, which was a kind gift from Jianming Qiu (Kansas University Medical Center). Infections were performed as described above.

### Western blot analysis.

Samples for Western blotting were prepared by lysing cells or an equivalent amount of input SV40 in RIPA buffer (50 mM Tris-HCl [pH 7.4], 1% NP-40, 0.25% sodium deoxycholate, 150 mM NaCl, 1 mM EDTA, 0.1% SDS). Samples were separated on a 10% SDS-PAGE gel. After transfer to a polyvinylidene difluoride (PVDF) membrane, samples were probed with a 1:1,000 dilution of anti-large T antigen (PAb108) or anti-VP1 (PAb 597) primary antibody and horseradish peroxidase-conjugated secondary antibody diluted in Tris-buffered saline (TBS) buffer with 0.1% Tween 20 and 5% nonfat milk, followed by chemiluminescence exposure and image acquisition with a FluorChem E Imager (ProteinSimple, CA).

### Indirect immunofluorescence.

CV-1 cells were seeded on 8-well chamber glass slides (Millipore, MA). At the indicated times after polyomavirus or lentivirus infection, cells were fixed with 3.7% paraformaldehyde (PFA) for 30 min and then permeabilized with 1% saponin for 30 min at room temperature, followed by primary and secondary antibody staining (each at a 1:100 dilution) and confocal microscopy analysis. Primary antibodies used were anti-large T antigen (PAb108) and anti-VP1 (PAb 597).

### Experiments with conditioned medium.

Conditioned medium was collected 72 h after mock infection or infection of CV-1 cells in a 24-well plate with wild-type SV40 at an MOI of 10. Conditioned medium was centrifuged for 5 min at 1,500 rpm, and the supernatant was added undiluted to ~5 × 10^4^ uninfected CV-1 cells, which in some cases had been previously transfected as above with siRNA targeting GM3 synthase. Based on flow cytometry of large T antigen expression in infected cells, we estimated that this results in an MOI of ~500. Cells were examined by phase-contrast microscopy 4 h later. For some experiments, conditioned medium was treated with 10 µM GM1 or a 1:2,000 dilution of neutralizing anti-SV40 antiserum (obtained from James Pipas) for 1 h at 37°C prior to addition to cells.

### GM1 supplementation assay.

CV-1 and Vero cells were either left untreated or treated with 10 µM GM1 overnight in DMEM with 1% FBS. The cells were then washed with regular cell culture medium, followed by one of the various indicated treatments.

### Luciferase assay.

The Gluc assay was performed using a BioLux *Gaussia* luciferase assay kit (New England Biolabs, MA) according to the manufacturer’s standard assay protocol. Briefly, CV-1 cells seeded on 8-well chamber glass slides were infected with wild-type BKpsV or the K68S mutant at 10^5^ genome copies/cell. Four hours later, medium was replaced with fresh medium lacking pseudovirus. At the indicated time postinfection, 20 µl of cell culture medium was analyzed for Gluc activity. A Veritas microplate luminometer (Promega, CA) with injectors was used to inject luciferase reaction solution and read the results (relative light units).

### SV40 VP1 pentamer expression, purification, and characterization.

Full-length wild-type SV40 VP1 and its derivative mutants were constructed with the pGEX-4T2 plasmid (GE Healthcare, Piscataway, NJ), expressed in *Escherichia coli* BL21(DE3) cells (Agilent) and purified by using glutathione *S*-transferase (GST) affinity purification and thrombin cleavage, as previously described (21). Mutant VP1 unable to form VLPs was truncated after amino acid position 305 (21).

For electron microscopy, purified pentamers were diluted in pH 7.2 PBS at 20 µg/ml and analyzed after negative staining with 1% uranyl acetate (21) via Tecnai T12 transmission electron microscopy at the Yale Electron Microscopy Core Facility. For transmission electron microscopy of infected cells, 2 × 10^6^ CV-1 cells were mock infected or infected with wild-type or A70L SV40 at an MOI of 1.5. At 64 h postinfection, samples were fixed, stained with 2% uranyl acetate, dehydrated, embedded, sectioned, and mounted onto copper grids as described elsewhere (40).

Purified pentamers were added to CV-1 cells maintained in DMEM containing 1% (vol/vol) FBS.

### Cross-linking truncated VP1 pentamers with antibody.

Aliquots (10 μg/ml) of truncated VP1 pentamers in DMEM without serum were incubated with a 1:100 dilution of nonneutralizing anti-VP1 PAb597 or with 1:100 PAb597 plus a 1:100 dilution of anti-mouse IgG for 30 min at 37°C prior to addition to uninfected CV-1 cells. Control cells were treated identically except without pentamer addition. Images were taken 4 h later.

### Treatment of cells with cholera toxin.

Cholera toxin subunit B (1-mg/ml stock solution in H_2_O) was diluted to a final concentration of 40 µg/ml and 100 µg/ml in culture medium and added to CV-1 cells. Images were obtained 24 h later.

## SUPPLEMENTAL MATERIAL

Figure S1  SV40 mutants defective for GM1 binding do not display a replication defect. (A) CV-1 cells were infected at an MOI of 10 with wild-type SV40 or a GM1-binding-defective mutant containing three mutations (A70L/F75L/H129Q) in VP1 (LLQ mutant). Results of a Western blot analysis of large T antigen (LTg) expression at the indicated time points postinfection are shown (top panel). The same blot was reprobed with anti-β-actin for the loading control. (B) Transmission electron micrographs of CV-1 cells 64 h after mock infection, infection with wild-type SV40, or with a GM1-binding-defective mutant (A70L) that fails to vacuolize (21). Note numerous virus particles in the nuclei of infected cells. Download Figure S1, TIF file, 2.4 MB

Figure S2  GM3 synthase knockdown does not block virus entry. CV-1 cells were transfected with GM3 synthase siRNA or a control RISC-free siRNA. At 24 h posttransfection, cells were infected with SV40 at an MOI of 10. After 24 h, cells were fixed and permeabilized with methanol, stained with anti-large T antigen antibody, and analyzed by flow cytometry. Download Figure S2, TIF file, 0.3 MB

Figure S3  An SV40 VP4 mutant affects virus release but not virus replication. CV-1 cells were infected with wild-type SV40 or a VP4 mutant SV40 at an MOI of 10. At 72 h postinfection, cell culture medium and cells were collected. Cells were lysed by freeze-thawing, and virus in the medium and the cell lysate was quantified by infecting naive CV-1 cells, staining for large T antigen, and flow cytometry. Download Figure S3, TIF file, 0.2 MB

Figure S4  SV40 replication is not initiated during the first 4 h of infection with conditioned medium. CV-1 cells were mock infected or infected with SV40. At 72 h postinfection, conditioned medium was collected and used to infect naive CV-1 cells. Four hours postinfection, cells were fixed, permeabilized, and stained with an anti-large T antigen (LTg) antibody (red) and DAPI (blue). Cells stained 72 h after infection with SV40 were used as a control. Cells were examined by fluorescence confocal microscopy. Download Figure S4, TIF file, 2.6 MB

Figure S5  Comparison of cell surface GM1 levels between Vero cells and CV-1 cells. Untreated CV-1 or Vero cells, or Vero cells treated with 10 µM GM1 for 16 h were stained with CTXB-fluorescein isothiocyanate (FITC) and analyzed by flow cytometry. Unstained, untreated Vero cells were used as a negative control. Download Figure S5, TIF file, 0.4 MB

Figure S6  Wild-type and K68S BKpsV infect CV-1 cells to a comparable extent. CV-1 cells were infected with 10^5^ genome equivalents per cell of wild-type or K68S BKpsV expressing Gluc. Cell culture medium was tested for *Gaussia* luciferase activity 4 h and 24 h after infection. Download Figure S6, TIF file, 0.2 MB

Figure S7  Analysis of VP1 pentamers. (A) Full-length SV40 VP1 (wild type), a full-length GM1 binding-defective LLQ mutant, and C-terminal deletion mutant of VP1 (truncated) were purified from *E. coli* cells by using GST affinity purification followed by thrombin cleavage. Purified proteins were analyzed by SDS-PAGE and staining with SimplyBlue SafeStain (Invitrogen). (B) Purified full-length, wild-type SV40 VP1 and truncated pentamers were stained with uranyl acetate and examined by electron microscopy. (C) Vacuole formation induced by wild-type and LLQ mutant pentamers was tested at 50 µg/ml as described in the legend for Fig. 5A. Download Figure S7, TIF file, 2.4 MB

Figure S8  Time course of pentamer-induced vacuole formation. CV-1 cells were treated with 10 µg/ml of the indicated pentamers. Phase-contrast micrographs were taken at the indicated times posttreatment. The top two rows are the same images as those shown in Fig. 5A. Download Figure S8, TIF file, 2.6 MB
