# Supplementary figures and images for: Interaction between Simian Virus 40 Major Capsid Protein VP1 and Cell Surface Ganglioside GM1 Triggers Vacuole Formation
Source: mBio. 2016 Mar 22;7(2):e00297-16. doi: 10.1128/mBio.00297-16 (PMC4807364; doi:10.1128/mBio.00297-16)

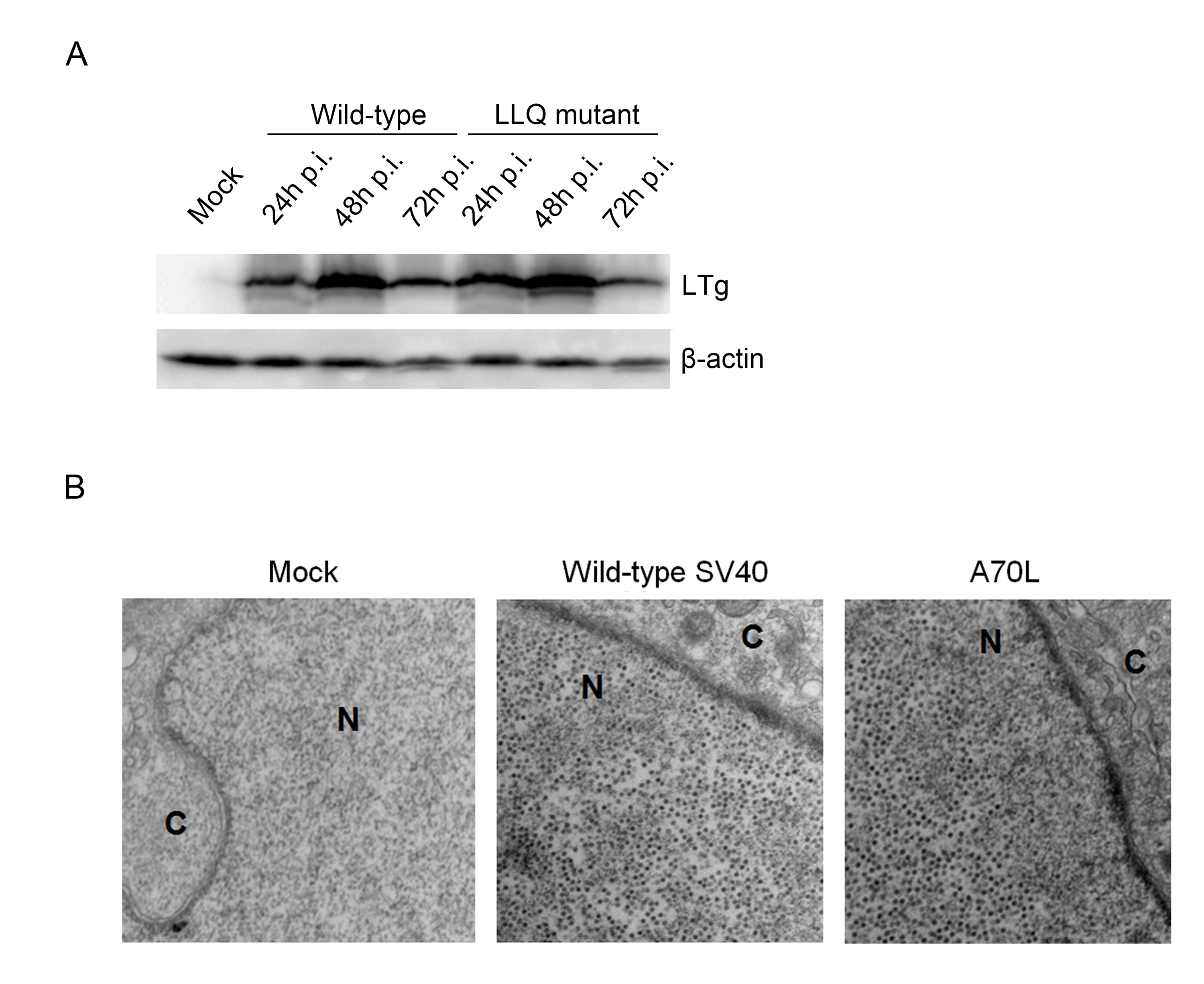

Supplement: Figure S1 — SV40 mutants defective for GM1 binding do not display a replication defect. (A) CV-1 cells were infected at an MOI of 10 with wild-type SV40 or a GM1-binding-defective mutant containing three mutations (A70L/F75L/H129Q) in VP1 (LLQ mutant). Results of a Western blot analysis of large T antigen (LTg) expression at the indicated time points postinfection are shown (top panel). The same blot was reprobed with anti-β-actin for the loading control. (B) Transmission electron micrographs of CV-1 cells 64 h after mock infection, infection with wild-type SV40, or with a GM1-binding-defective mutant (A70L) that fails to vacuolize (21). Note numerous virus particles in the nuclei of infected cells. Download [file mbo002162740sf1.tif]

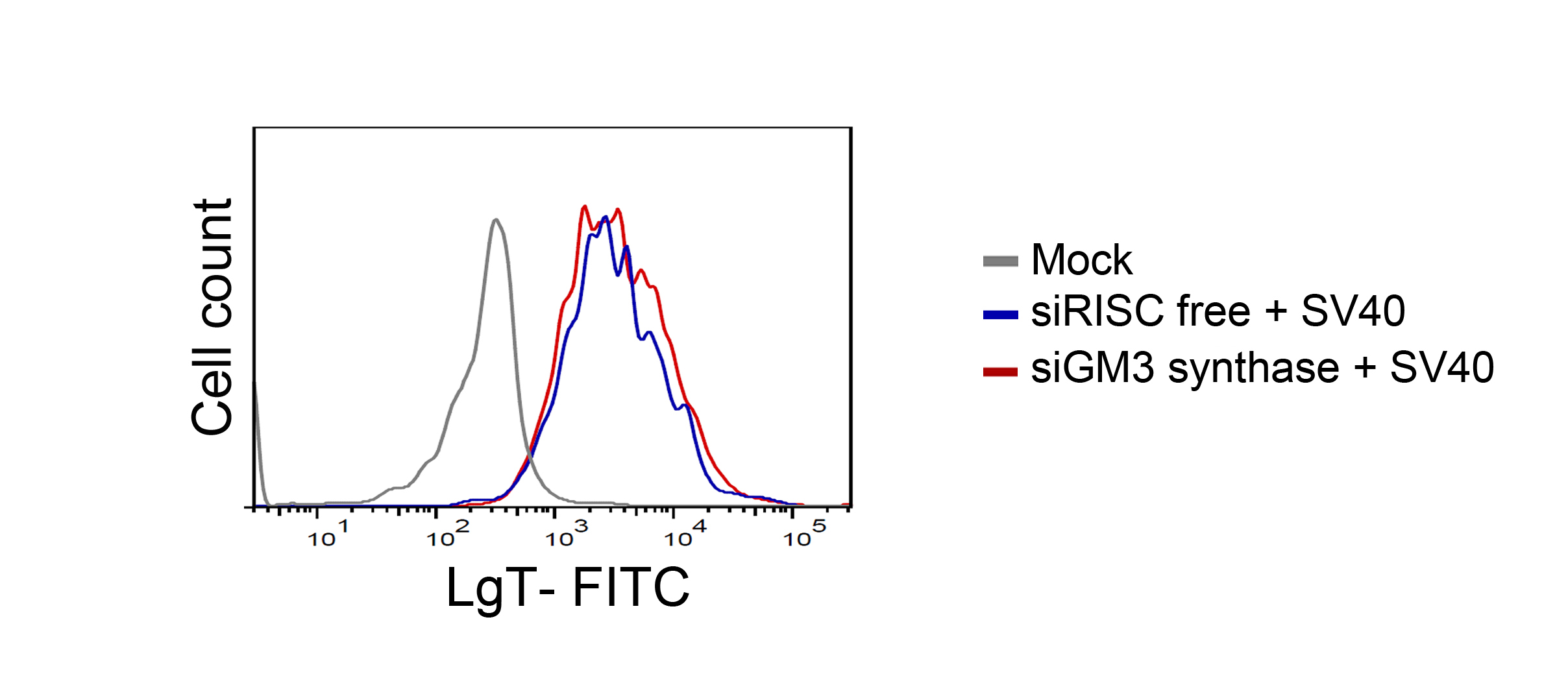

Supplement: Figure S2 — GM3 synthase knockdown does not block virus entry. CV-1 cells were transfected with GM3 synthase siRNA or a control RISC-free siRNA. At 24 h posttransfection, cells were infected with SV40 at an MOI of 10. After 24 h, cells were fixed and permeabilized with methanol, stained with anti-large T antigen antibody, and analyzed by flow cytometry. Download [file mbo002162740sf2.tif]

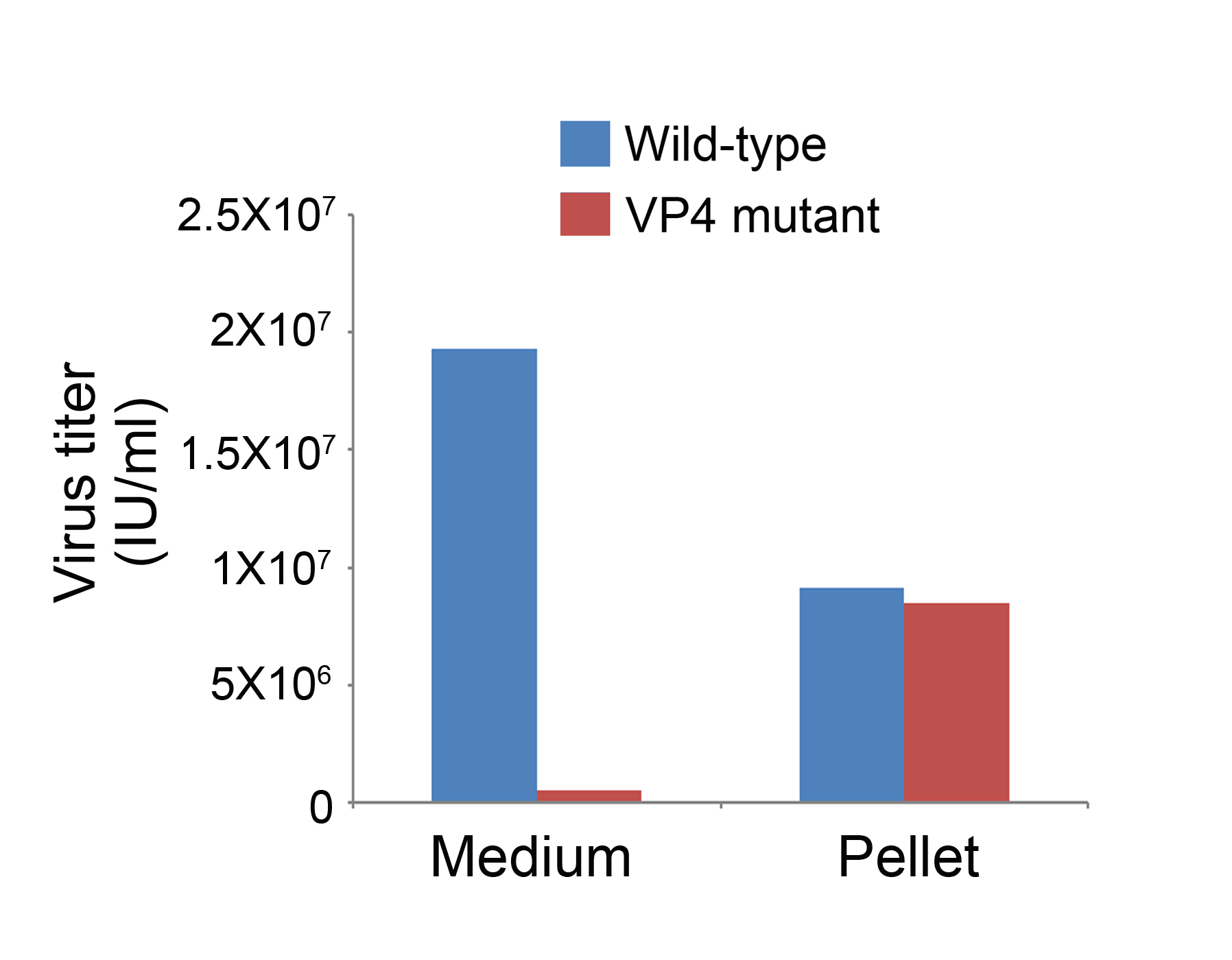

Supplement: Figure S3 — An SV40 VP4 mutant affects virus release but not virus replication. CV-1 cells were infected with wild-type SV40 or a VP4 mutant SV40 at an MOI of 10. At 72 h postinfection, cell culture medium and cells were collected. Cells were lysed by freeze-thawing, and virus in the medium and the cell lysate was quantified by infecting naive CV-1 cells, staining for large T antigen, and flow cytometry. Download [file mbo002162740sf3.tif]

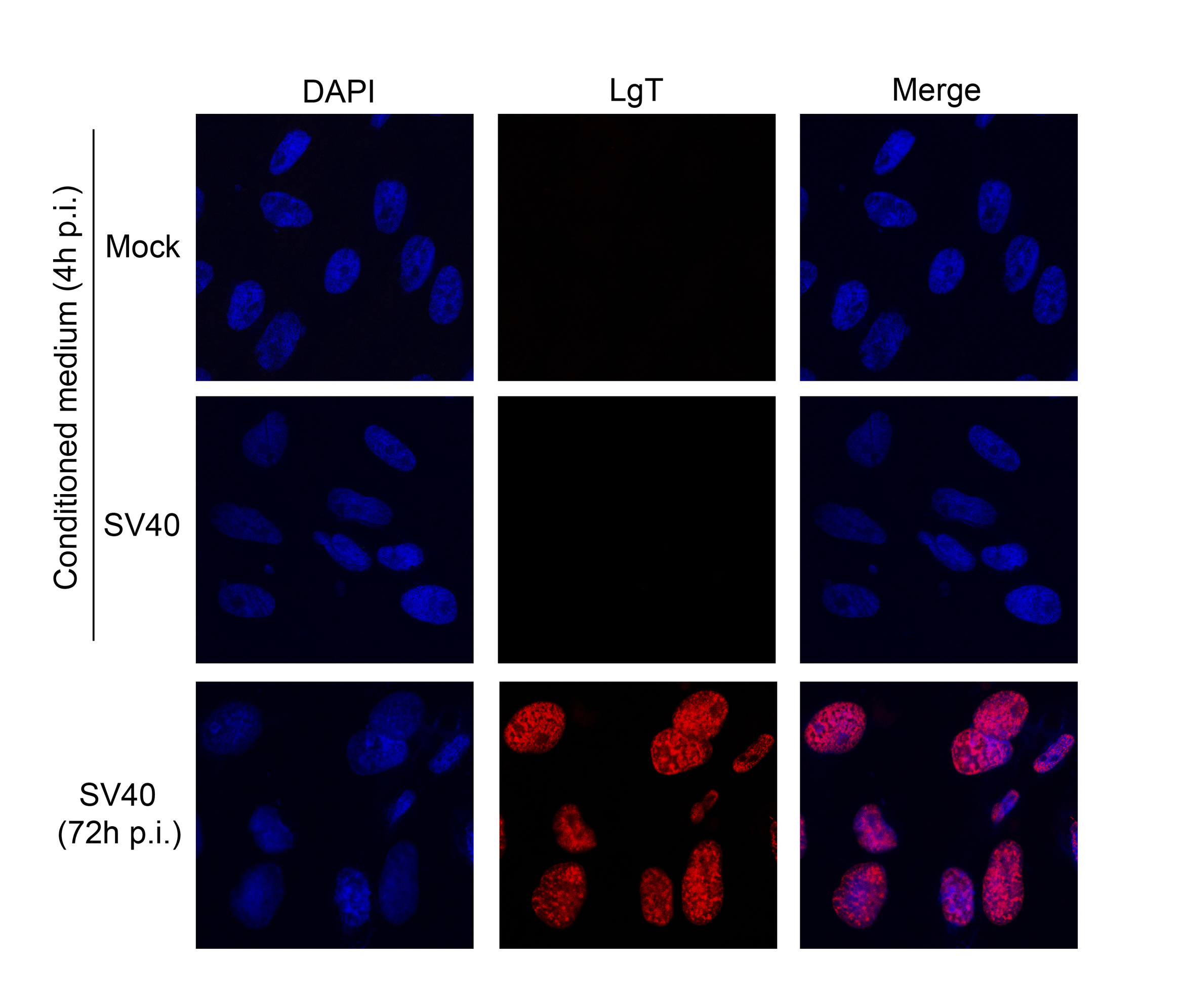

Supplement: Figure S4 — SV40 replication is not initiated during the first 4 h of infection with conditioned medium. CV-1 cells were mock infected or infected with SV40. At 72 h postinfection, conditioned medium was collected and used to infect naive CV-1 cells. Four hours postinfection, cells were fixed, permeabilized, and stained with an anti-large T antigen (LTg) antibody (red) and DAPI (blue). Cells stained 72 h after infection with SV40 were used as a control. Cells were examined by fluorescence confocal microscopy. Download [file mbo002162740sf4.tif]

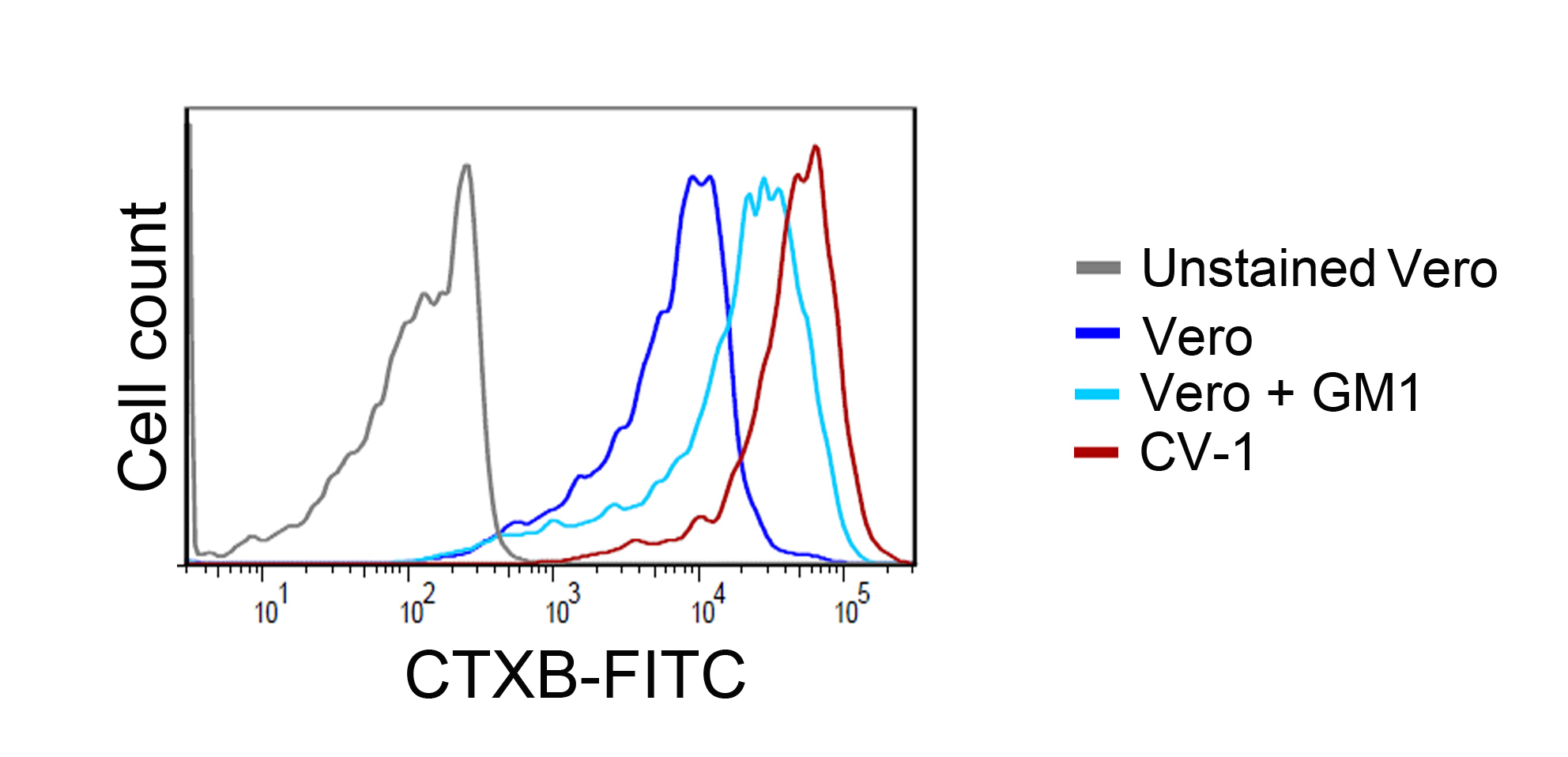

Supplement: Figure S5 — Comparison of cell surface GM1 levels between Vero cells and CV-1 cells. Untreated CV-1 or Vero cells, or Vero cells treated with 10 µM GM1 for 16 h were stained with CTXB-fluorescein isothiocyanate (FITC) and analyzed by flow cytometry. Unstained, untreated Vero cells were used as a negative control. Download [file mbo002162740sf5.tif]

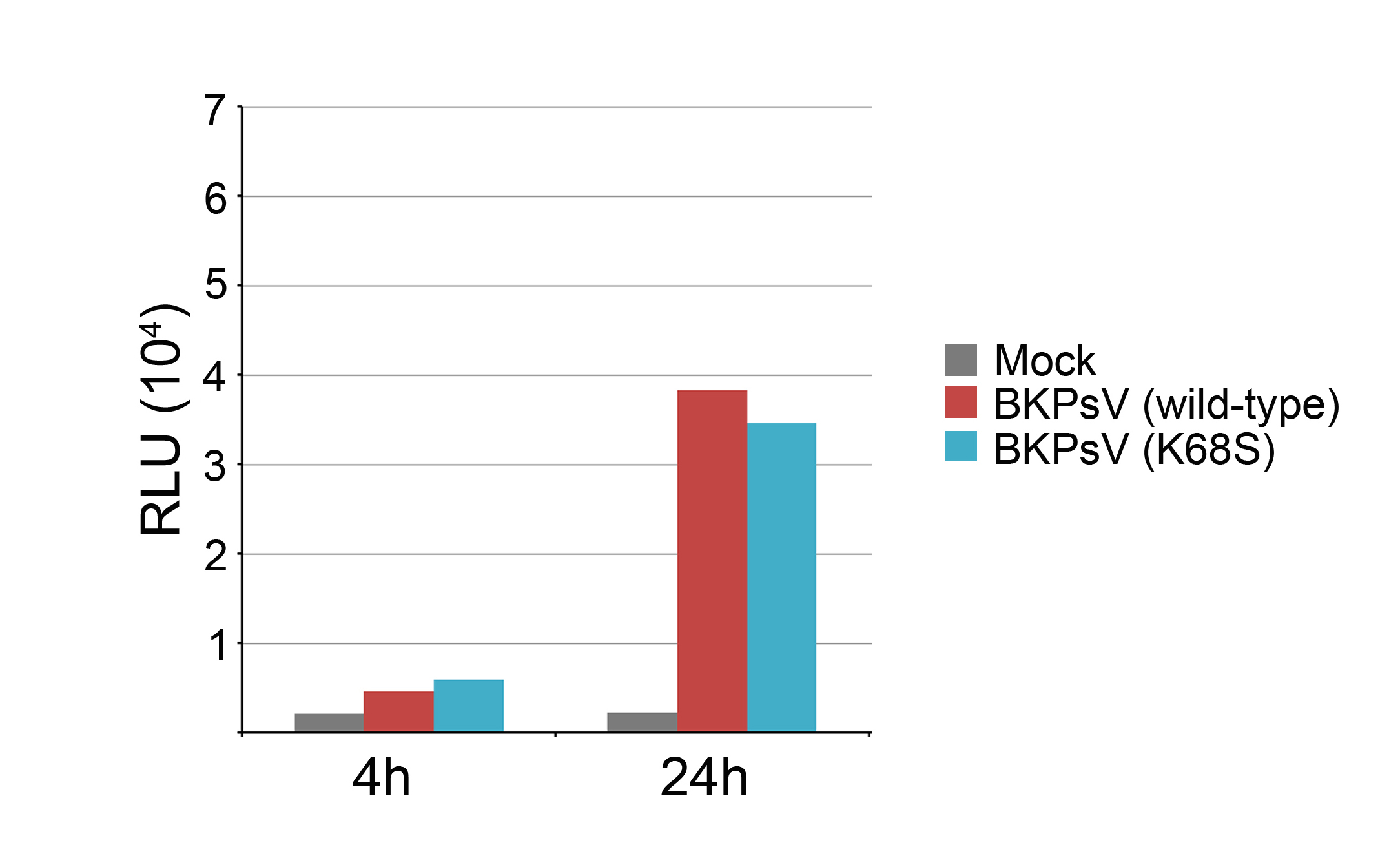

Supplement: Figure S6 — Wild-type and K68S BKpsV infect CV-1 cells to a comparable extent. CV-1 cells were infected with 105 genome equivalents per cell of wild-type or K68S BKpsV expressing Gluc. Cell culture medium was tested for Gaussia luciferase activity 4 h and 24 h after infection. Download [file mbo002162740sf6.tif]

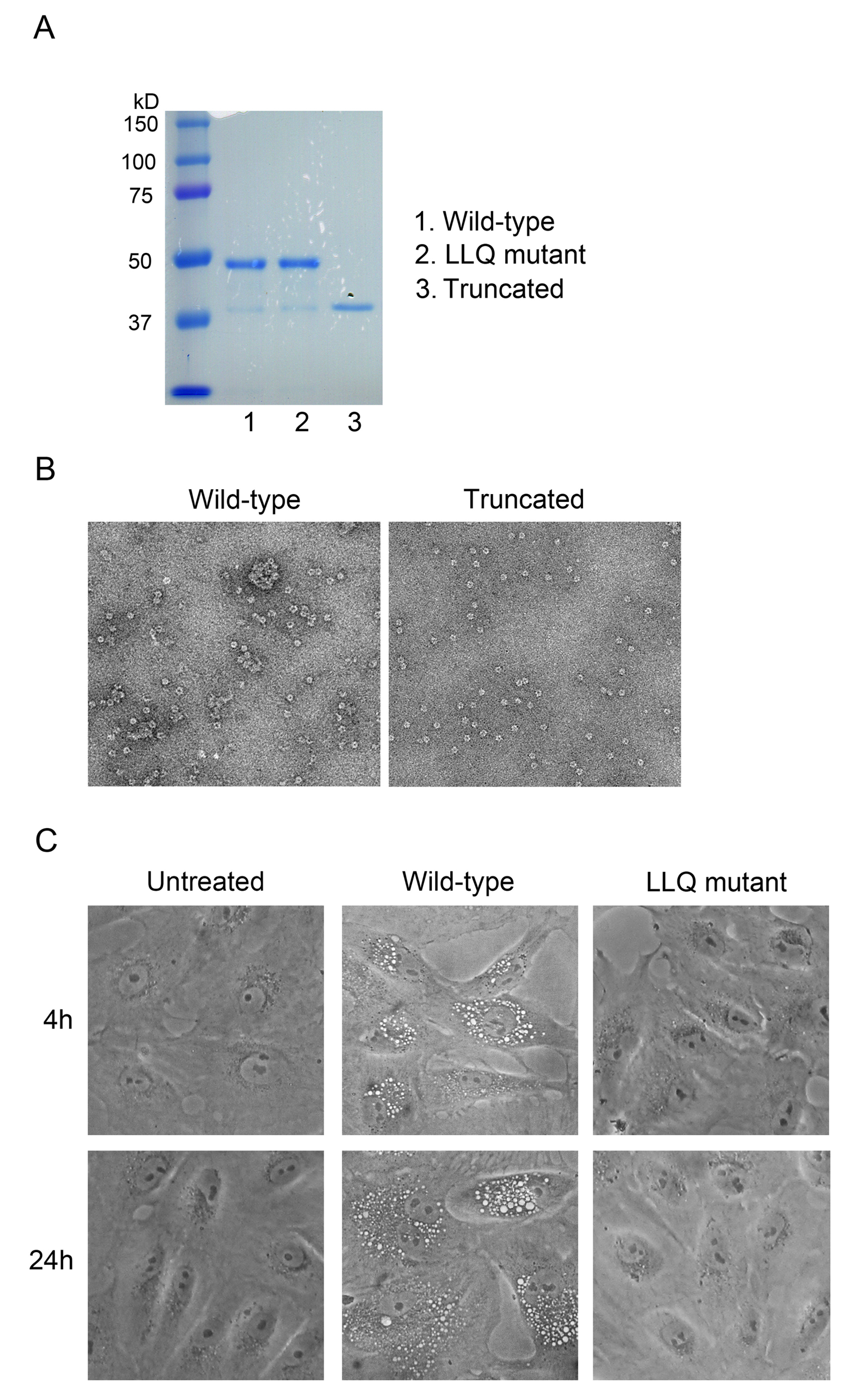

Supplement: Figure S7 — Analysis of VP1 pentamers. (A) Full-length SV40 VP1 (wild type), a full-length GM1 binding-defective LLQ mutant, and C-terminal deletion mutant of VP1 (truncated) were purified from E. coli cells by using GST affinity purification followed by thrombin cleavage. Purified proteins were analyzed by SDS-PAGE and staining with SimplyBlue SafeStain (Invitrogen). (B) Purified full-length, wild-type SV40 VP1 and truncated pentamers were stained with uranyl acetate and examined by electron microscopy. (C) Vacuole formation induced by wild-type and LLQ mutant pentamers was tested at 50 µg/ml as described in the legend for Fig. 5A. Download [file mbo002162740sf7.tif]

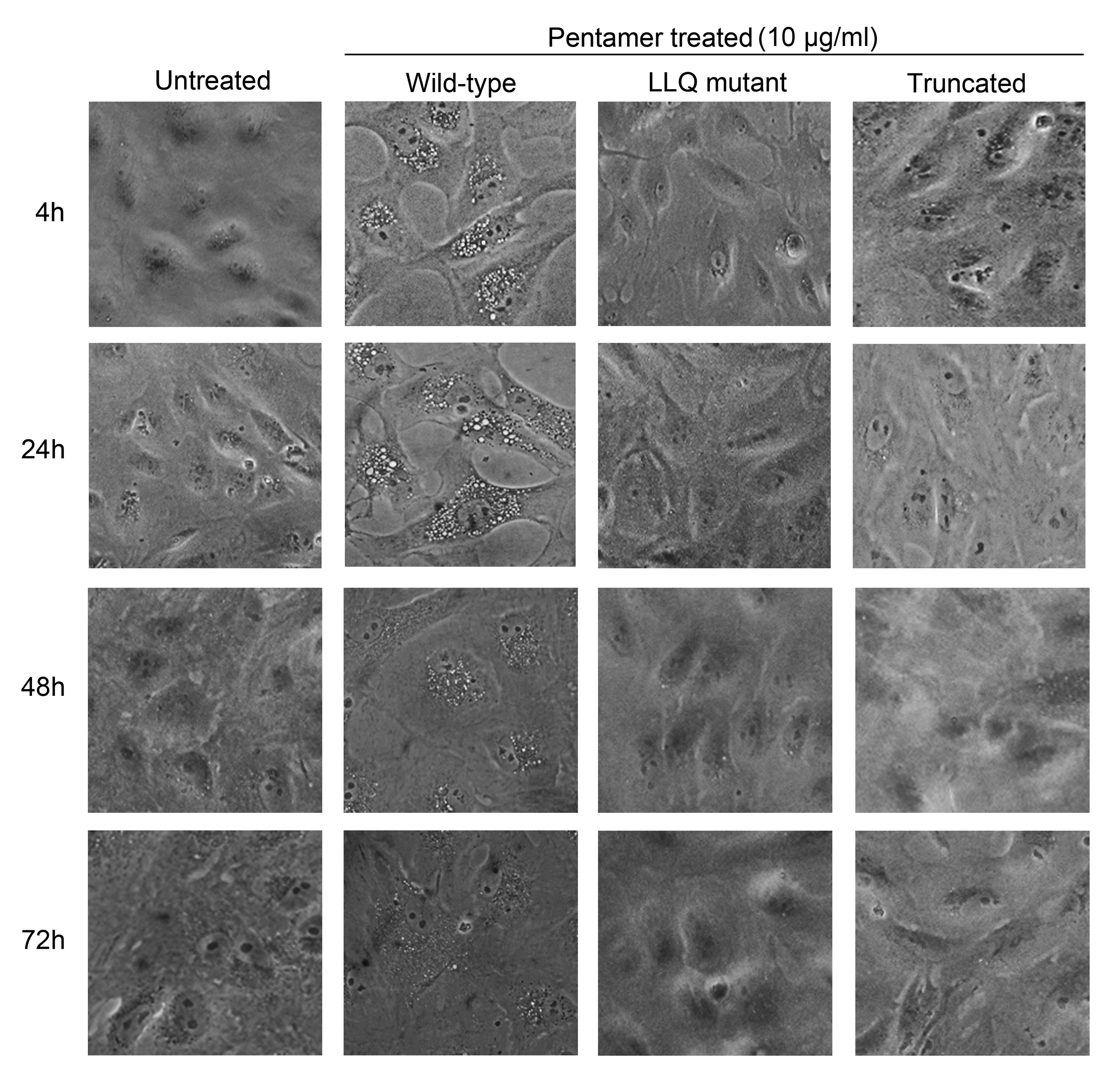

Supplement: Figure S8 — Time course of pentamer-induced vacuole formation. CV-1 cells were treated with 10 µg/ml of the indicated pentamers. Phase-contrast micrographs were taken at the indicated times posttreatment. The top two rows are the same images as those shown in Fig. 5A. Download [file mbo002162740sf8.tif]
